# Light-Emitting Lanthanide Periodic Mesoporous Organosilica (PMO) Hybrid Materials

**DOI:** 10.3390/ma13030566

**Published:** 2020-01-24

**Authors:** Anna M. Kaczmarek, Pascal Van Der Voort

**Affiliations:** COMOC–Center for Ordered Materials Organometallics and Catalysis, Department of Chemistry, Ghent University, Krijgslaan 281-S3, B-9000 Ghent, Belgium; pascal.vandervoort@ugent.be

**Keywords:** periodic mesoporous organosilica (PMO), hybrid materials, light emission, light harvesting, lanthanides, luminescence, sensing

## Abstract

Periodic mesoporous organosilicas (PMOs) have a well ordered mesoporous structure, a high thermal and mechanical stability and a uniform distribution of organic functionalities in the pore walls. The organic groups allow PMOs to be modified and functionalized by using a wide range of organic reactions. Since their first report in 1999, PMOs have found a vast range of applications, such as for catalysis, adsorbents, low-*k* films, biomedical supports and also for optical applications. Optical applications are very interesting as PMOs offer the possibility of designing advanced luminescent hybrid materials comprising of organic components, yet with much higher stability and very good processability. Despite their promising possibilities, the optical properties of pristine PMOs and PMOs grafted with d-metal or f-metal ions and complexes have been explored less frequently. In this review, we aimed to overview the exciting light emitting properties of various reported lanthanide PMO hybrid materials and interest the reader in this promising application for lanthanide PMO materials.

## 1. Introduction

Periodic Mesoporous Organoslicas (PMOs) are ordered templated mesoporous organosilicas, prepared by employing a surfactant as the template and a bridged silylated precursor [1]. PMOs were first reported in 1999 [2,3,4]. To date, they have found a wide range of applications, such as for catalysis, adsorbents, low-*k* films, chromatographic phases, biological/biomedical supports and for optical applications [5]. The wide range of applications is due to their well ordered mesoporous structure, high thermal and mechanical stability and uniform distribution of organic functionalities in their pore walls, as well as the huge variety of these functional groups. The properties of PMOs are driven by the organic functionalities embedded in the walls or grafted to the framework and are only limited by the imagination and skills of the scientist [6]. A very exciting and emerging application in the field of PMOs is optical applications. This can be based on PMOs baring luminescent groups or PMOs functionalized with metal complexes. Interesting work has been done on the topic of lanthanide grafted PMOs, which have already shown possible applications in white light generation or temperature and ion sensing and hold many exciting possibilities for the future. To the best of our knowledge, to date, there has been no review overviewing the current research work, which has been carried out concerning lanthanide PMOs and their applications.

In the introduction of this review, it should be pointed out that there is a common confusion and misunderstanding in the scientific literature between PMO materials and organically modified mesoporous silica. Such materials are much easier to prepare, but do not take advantage of the highest possible organic loading of the fluorophore. According to the most restricted definition, a PMO is a structure based only on silsesquioxanes, which means that the synthesis must be performed in the absence of a silica source (e.g., TEOS). Moreover, a sufficient porosity of the materials, typically obtained by structure-directing agents, must be obtained in order for it to be referred to as a PMO material. This review overviews materials, which are considered in literature as PMO materials and does not follow the very strict definition of PMOs. Moreover, a mixed PMO-type material exists, where a simple, non-bulky PMO precursor is used as a diluting agent for a bulky precursor. This enables obtaining ordered materials despite the use of bulky precursors (only at a low percentage). Such materials will be referred to in the text as ‘PMOs’ as they do not fulfill the strict definition of this class of materials.

## 2. Luminescence Properties Originating from PMOs Bearing Luminescent Organic Groups

This review concerns the topic of lanthanide PMO materials. However, it is impossible to talk about lanthanide PMO materials without introducing PMOs bearing fluorescent organic groups, which, most often, can potentially act as chromophores for lanthanide PMOs. The phrase “potentially act” has been used as some of these chromophore groups do not have binding sites to which a lanthanide ion could be bonded and the precursors would have to be modified for this. The reader is referred to the highlight paper published in 2009 by Inagaki et al. entitled “Luminescent periodic mesoporous organosilicas”, which overviews the most important findings in the field up until 2009 [7]. Here, we once again have a look at these findings as well as reviewing the more recent literature.

**Figure 1:**
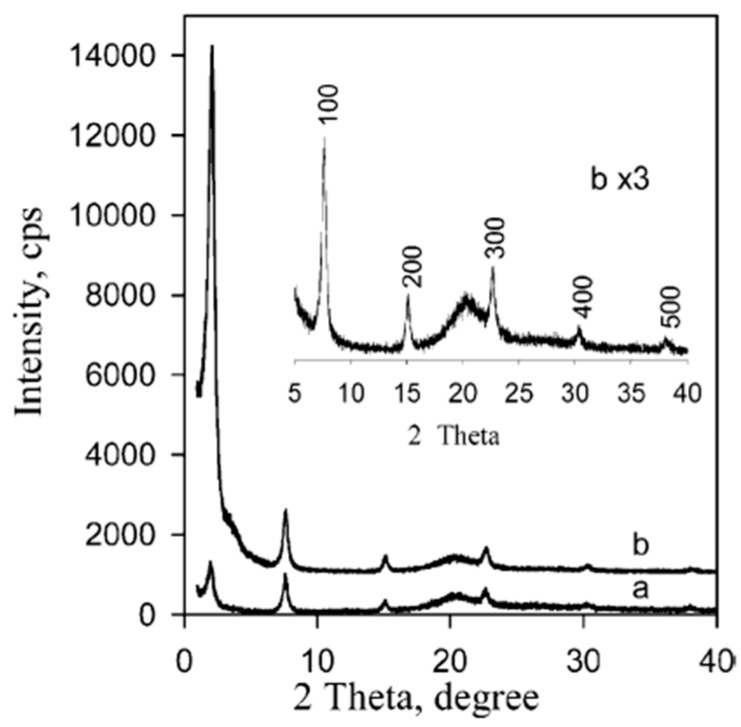
Figure of the PXRD profiles of the biphenylene-bridged periodic mesoporous organosilicas (PMO) is presented. (**a**) The as-made material containing surfactants, (**b**) the surfactant-free material obtained by solvent extraction [9].

We already know that the properties of PMOs are driven by the organic functionalities embedded in the walls or grafted to the framework and are only limited by the imagination (and skills) of the researcher [6]. Here, we focus on the detailed analysis of PMOs baring fluorescent groups. Sometimes, PMOs prepared from 100% chromophore precursors can suffer from solid-state quenching; therefore, often, a better approach from the luminescence point of view is preparing PMOs by co-condensation, which results is a high loading of the organic groups but avoids concentration quenching. In 2002, Inagaki et al. reported on a surfactant-mediated ordered benzene-silica PMO material [8]. The material had a hexagonal array of mesopores with a lattice constant of 52.5 Å and crystal-like pore walls that exhibited structural periodicity with a spacing of 7.6 Å along the channel direction. PXRD confirmed that the material had both a mesoscale (*d* = 45.5, 26.0, and 22.9 Å) and molecular-scale (*d* = 7.6, 3.8 and 2.5 Å) periodic structure. The periodic pore surface structure resulted from the alternating hydrophilic and hydrophobic layers, composed of silica and benzene, respectively. Through N_2_ adsorption isotherms, the existence of uniform mesopores was confirmed. The BET (Brunauer–Emmett–Halenda) surface area and mesopore volume were 818 m^2^/g and 0.66 cm^3^/g. Soon afterwards, Inagaki et al. also communicated on a biphenylene-bridged PMO with molecular-scale periodicity in the pore walls [9]. This is a second example of a potentially fluorescent PMO, although, back then, these materials were not investigated for this property. The biphenylene-bridged PMO was prepared employing the 4,4′bis(triethoxysilyl)biphenyl [(C_2_H_5_O)_3_Si-(C_6_H_4_)_2_-Si(OC_2_H_5_)_3_] precursor. It was reported that in order to obtain a highly ordered material it was important to control the reaction conditions and initial reactant ratio. The PXRD of the material exhibited a strong peak at 2θ = ca. 2.0°, suggesting a mesoscopically ordered structure. Additionally, at medium scattering, five angled peaks with a *d* spacing of 11.6, 5.9, 3.9, 2.9, and 2.4 Å were found (Figure 1). The peaks were assigned as a periodicity with spacing of 11.6 Å, which is larger than the periodicity observed in the benzene-bridged PMO (7.6 Å). This is due to the larger length of the biphenylene compared to the benzene groups. The BET surface area and mesopore volume were 869 m^2^/g and 1.15 cm^3^/g, respectively.

**Figure 2:**
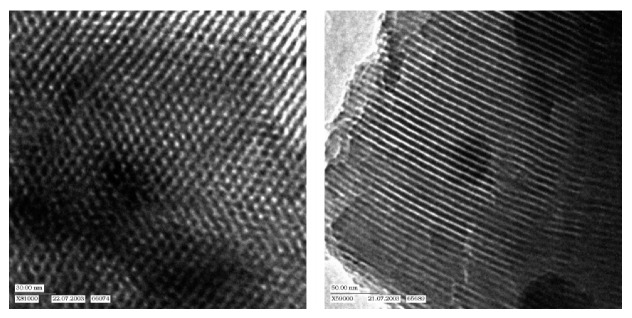
Representative TEM images of a mesoporous thiophene-bridged organosilica material (view parallel (**left**) and perpendicular (**right**) to the pore axes [13].

Two years later, Ozin et al. reported on a PMO prepared by polymerizing phenylene-bridged silsesquioxane precursors containing an incremental increase in methylene spacers [1,4-(CH_2_)_n_C_6_H_4_ (n = 0–2)] [10]. Reports on other PMOs, built from chromophore groups in the pore walls followed in the following years. For example, Fröba et al. reported on a PMO with crystal-like pore walls synthesized from the 1,4-bis-((*E*)-2-(triethoxysilyl)vinyl)benzene (BTEVB) [11]. This precursor was obtained via Pd-catalized double Heck coupling of 1,4-dibromobenzene with vinyltriethoxysilane. Moreover, Fröba et al. reported the first example of a thiophene-bridged PMO, which had very large pores (5–6 nm) [12]. Around the same time, more detailed investigations were also being carried out on the influence of the surfactant in the synthesis procedure. For example, Wang et al. investigated the synthesis of a PMO from the bis(triethoxylsilylethen-2-yl)benzene ((C_2_H_5_O)_3_Si-CH=CH-C_6_H_4_-CH=CH-Si(OC_2_H_5_)_3_), which is the same precursor as that used for the PMO described above [13]. When no surfactant was used, the PXRD of the materials proved the presence of diffraction peak at 11.7, 5.9, 3.9 and 2.95 Å, which is consistent with the occurrence of the molecular order of the bis (ethane-2-yl) benzene moieties. The TEM images clearly reveal the presence of a hexagonal arrangement of the well-defined pores with uniform dimensions (Figure 2). However, no low-angle diffraction peak was observed, indicating the absence of a mesoscopic structure.

In 2007, Bhaumik et al. reported on a diimine-incorporated PMO, where a group functioning as a tunable chemosensor was covalently grafted into the mesopore walls [14]. To obtain this PMO, first, a Schiff-base-bridged fluorescent precursor was obtained. This was done in a reaction between 2,6-diformyl-4-methylphenol and (3-aminopropyl)triethoxysilane. The obtained PMO showed uniform mesopores and considerably high BET surface areas (412 m^2^/g) and displayed a tunable chemosensor property in the solid state. The materials exhibited very strong photoluminescence, with an emission spectrum around 500–550 nm. Thanks to this property, the material could be employed as a possible optical sensor for the detection of metal cations (Fe^3+^, Zn^2+^). Apart from chemosensors, the unique photophysical properties of the PMO material could possibly be utilized in light-harvesting and optoelectronic applications.

**Figure 3:**
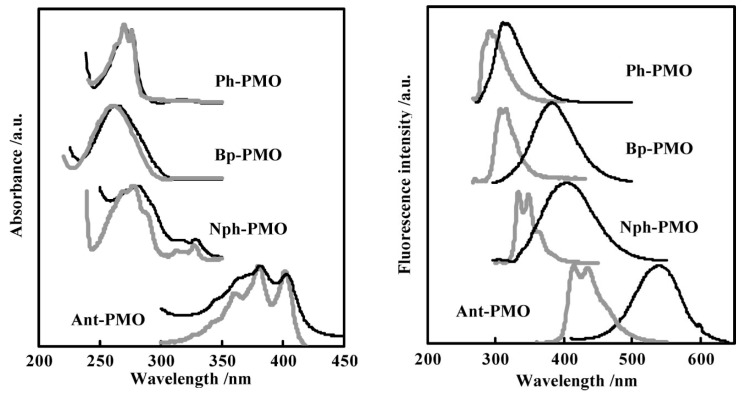
(**Left**): Absorption spectra of the benzene (Ph)-, biphenyl (Bp-), naphthalene (Nph)- and anthracene (ant)-PMO films and their precursors in 2-propanol (around 10^−5^ M). (**Right**): Fluorescent spectra of the same PMO films and their precursors also in in 2-propanol [18].

Around the years 2007–2009, a clear interest in the use of PMOs built from aromatic groups for light-harvesting and optoelectronic applications emerged. Leyva et al. reported on an electroluminescent ‘PMO’ containing 9,10-diarylanthracene units [15]. This is, however, not a real PMO material, but a mixture of the silane with TEOS and does not take advantage of the highest possible organic loading of the fluorophore. They showed that the use of a structure-directing agent (comparing to amorphous organosilica materials) plays a role shifting to the emitted light to the blue part of the spectrum with only slightly decreasing the efficiency. These results show that it is possible to use PMOs for the preparation of OLEDs, which could be used in commercial applications. Ha et al. published work on a fluorescent ‘PMO’ prepared from tetraethoxysilane (TEOS) and carbazole modified silica precursor [16]. According to the strict definition of PMO materials, this is not a PMO, as it is prepared in the presence of a silica source (TEOS). The carbazole precursors exhibited a blue shift in the excitation spectrum compared to pure carbazole. Fröba et al. reported, in 2008, on a systematic extension of the length of the organic conjugated π-system of organic-inorganic silica materials [17]. For this purpose, two new bis-silylated compounds with 18 p-electron systems were synthesized: 4,4′-bis((*E*))-2-(triethoxysilyl)vinyl)stilbene and 1,2-bis(4-((*E*)-2-(triethoxysilyl)vinyl)phenyl)diazene. However, with these extended precursors, it was not possible to obtain real PMO materials (no mesoscale ordering observed in the PXRD and TEM). Yet, the authors showed, for the first time, that it was possible to obtain colored mesoporous hybrid materials from one single bis-silylated silica source. In the same year, Inagaki et al. reported on several new PMOs prepared from 100% organosilica precursors containing bridging organics of 1,4-phenylene, 4,4′-biphenylylene, 2,6-naphtylene and 9,10-anthrylene [18]. For all the composites, transparent films with periodic mesostructures were successfully obtained. The fluorescence of the PMO thin films was investigated. The fluorescence spectra of the PMO films showed a significant red-shift and also broadening of the peaks compared to those of their precursor solutions, suggesting excimer formation (Figure 3). The quantum yields (QYs) of the precursors and films were recorded. It was observed that the QYs of the PMO films were lower than those of their precursors. Only for the 4,4′-biphenylylene PMO, the QY increased above that of the precursor, in spite of the excimer formation. The high absorption coefficient (87,000 cm^−1^) and high QY (45%) of the 4,4′-biphenylylene-based PMO film shows its great potential for use as a fluorescent material.

In the work of He et al., the successful incorporation of the photoluminescent perylene-bridged silsesquioxane into the periodic pores of PMO films is presented [19]. Despite the large and bulky organic ligand, the ‘PMO’ showed well organized hexagonal mesophases in the PXRD, which was consistent with TEM measurements. The well organized hexagonal mesophases were possible to obtain with such a bulky linker because a mixed PMO was prepared in which the bulky ligand was not used to construct the whole PMO framework, but a mixture of 1,2-bis(triethoxysilyl)ethane and perylene-bridged silsesquioxane was employed). The ‘PMO’ showed fluorescence in the 550–700 nm range with a maximum of around 600 nm. One year later, Inagaki et al. published a work comparing the fluorescence emission from 2,6-naphtylene-bridged PMO with an amorphous or crystal-like framework [20]. In the crystal-like framework, the PMO exhibited sharp emission spectra attributed to a monomer band. For the amorphous PMO excimer band, emission was observed. These findings suggest that the naphthalene moieties fixed within the crystal-like framework are isolated in spite of their very densely packed structure. These findings suggest that the interactions, and therefore, also the optical properties, of the organic moieties embedded in the PMO frameworks are controlled by arranging the inorganic/organic hybrid structures. Moreover, in 2009, Inagaki et al. reported on the synthesis and optical properties of the 2,6-anthracene-bridged PMO [21]. The reported PMO showed absorption at near the ultraviolet region and blue-green fluorescence with a QY of 13–15%. The authors suggested that the visible emitting 2,6-anthracene-bridged PMO could be promising as a phosphor material for LEDs. Okada et al. reported that the biphenyl PMO can be used for light harvesting applications [22]. They showed that highly efficient energy transfer from biphenyl in the PMO framework to a small amount of coumarin 1 dye doped in the mesochannels is present. It was estimated that light energy absorbed by approximately 125 biphenyl groups from the PMO framework was funneled to a single coumarin 1 molecule in the channel with almost 100% quantum efficiency. This resulted in a significant increase in emission of the coumarin 1 dye. These results show the great potential of such PMOs with aromatic moieties in the framework as light-harvesting scaffolds for light-emitting devices and photoreaction systems. Additionally, it was highlighted in the work that the absorption wavelength of the PMO can be tuned by appropriate selection of framework organic groups. To give an example, anthracene-based PMO or oligo (phenylenenvinylene)-based PMO have been shown to exhibit strong absorption bands around 400 nm, which may prove to be very good for solar energy applications. The downside of this system is that biphenyl-PMO has the limitation of absorbing only UV light, whereas visible-light-harvesting would be very important for numerous applications. Shortly after, Inagaki et al. reported on a visible-light-harvesting 9(10*H*)-acridone bridged PMO [23]. This PMO exhibited efficient light-harvesting antenna properties for visible light, at wavelengths up to 450 nm. In this work, the PMO was doped with DCM dye (4-dicyanomethyle-2-methyl-6-*p*-dimethyaminostyryl-4*H*-pyran). This dye was selected for the study as its absorption band overlaps well with the emission band of the 9(10*H*)-acridone bridged PMO. In the experiments carried out, it was observed that the PMO emission was gradually quenched and strong emission of the DCM dye was alternatively observed (at around 600–630 nm). This indicated that successful energy transfer took place between the acridone framework and the DCM dye. It should also be noted that the authors reported that the fluorescence QY of the PMO doped with DCM was higher than that of the pristine PMO because of direct excitation energy transfer from acridone to DCM dye molecules without a radiation-reabsorption process.

**Figure 4:**
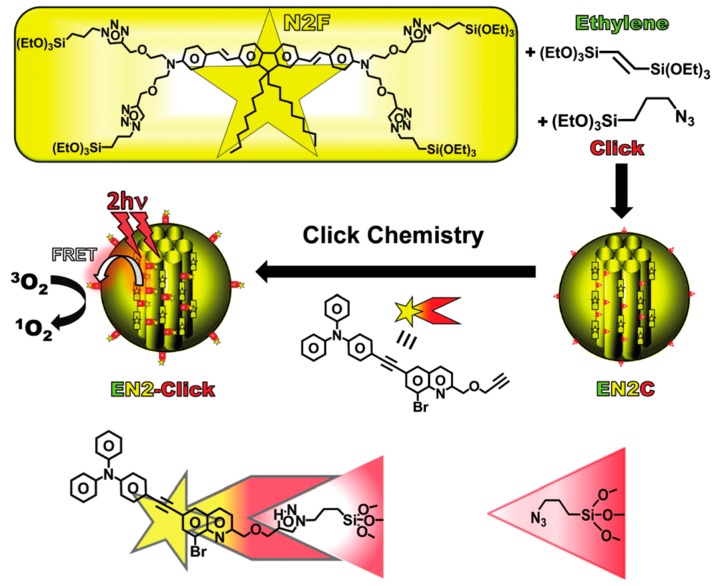
Representation of the design of the ethenylene-azidopropyl-bridged ‘PMO’ nanoparticles functionalized with a propargylated fluorescent bromo-quinoline photosensitizer for PDT applications [35].

After these important publications, scientists put energy into developing PMOs with aromatic moieties which were further grafted with metal ions, both d-metals and f-metals. This review focuses solely on the work carried out of f-metal grafted PMOs. However, the reader is referred to interesting studies on d-metal grafted PMOs, for example, those given in the references [24,25,26,27,28,29]. Despite the growing interest in metal grafted PMOs for obtaining optical properties, some recent studies on PMOs containing aromatic moieties for optical applications can still be found in literature. For example, in 2012, Inagaki et al. published work in which the fluorescent bipyridine receptor with two silyl groups was synthesized and covalently attached to the pore walls of biphenyl-bridged PMO powder [30]. It was observed that the fluorescent intensity from the bipyridine receptor was highly enhanced by the light-harvesting properties of the biphenyl PMO. This PMO was investigated for its behavior in the presence of metal ions. The enhanced emission of the bipyridine receptor was quenched when a low concentration of Cu^2+^ was added. In addition, upon the addition of Zn^2+^ ions, systematic changes in the fluorescence spectra (both excitation and emission) were observed. These results proved that PMOs may be interesting materials for use as fluorescent chemosensors. It is also worth mentioning that in a very different approach, photoluminescent PMOs were obtained by the group of Ozin [31]. They successfully incorporated photoluminescent silica (ncSi) into a PMO through a creative design of the precursor with an oligomeric capping ligand. This material showed multifunctional properties suitable for its use in optoelectronic and biomedical applications. In another study, Yang et al. reported on the synthesis of 1,10-phenantroline functionalized PMO, which worked as a metal ion sensor based on its photoluminescence properties [32]. This PMO did not show a very high BET surface area (up to 328 m^2^/g); however, it showed good ordering confirmed by PXRD and TEM images. The PMO showed different fluorescence response to different metal ions, with almost complete fluorescence turn off at higher concentrations of Cu^2+^ ions (10^−2^ M). This work once again proved that PMO materials are excellent candidates for ion sensing publications. Moreover, in the work of Ha et al., a PMO bearing ethidium bromide moieties in the framework was employed for ion sensing [33]. It showed selective monitoring of Hg^2+^ and Fe^3+^ ions in water as well as living cells (showing turn off fluorescence). TEM and SEM analysis showed that the material formed particles 50–350 nm in size, which makes them very suited for sensing applications. The PMO was sufficiently stable under biological conditions and could be used to monitor Hg^2+^ and Fe^3+^ ions in a wide range of pH. The authors reported that the fluorescence as well as color change behavior of the PMO was retrieved upon the addition of EDTA to the suspension. Fluorescent microscopy results showed that the ethidium bromide PMO had potential use in examining the toxicity or biocompatibility of Hg^2+^ and Fe^3+^ ions in living cells under either in vitro or in vivo conditions. In the work of Yu et al., an interesting strategy was proposed to integrate aggregation-induced emission (AIE) and aggregation caused quenching (ACQ) chromophores in the same PMO material [34]. According to the strict definition mentioned in the introduction, this is not really a PMO material as the framework is constructed from a silica source—3-aminopropyltriethoxysilane and 1,2-bis[4-(bromomethyl)phenyl]-1,2-diphenylethene and not purely of the 1,2-bis[4-(bromomethyl)phenyl]-1,2-diphenylethene linker. AIE and ACQ are important for certain fluorescence-based applications; however, they cannot easily collaborate as they are opposite luminescence behaviors. Here, a tetraphenylethene-bridged ‘PMO’ (AIE behavior) was used to host RhB dyes (ACQ behavior). This allowed fine-tuning the emission over the entire visible spectrum both in solid as well as film states. White light was obtained from the material (x = 0.32, y = 0.33) and a high QY = 49.6%. The authors showed that due to their high stability and solution processability, this ‘PMO’ hybrid material could be used in solid-state lighting and bioimaging applications. It should also be mentioned that the ‘PMO’ material was obtained in the form of nano-sized spherical particles (around 100–150 nm). More recently, in 2016, Jean-Olivier Durand described the synthesis of ethenylene-azidopropyl-bridged ‘PMO’ nanoparticles (~100 nm) (Figure 4) [35]. This PMO belongs to the class of mixed PMOs as it is formed in the co-condensation of bis)triethoxysilyl)-ethane with 3-azidopropyltriethoxysilane. The authors showed that the ‘PMO’ platform could be employed for loading and pH-triggered release of drugs. Employing click chemistry, the ‘PMO’ nanoparticles could be further post-functionalized with a novel fluorophore. The propargylated fluorescent bromo-quinoline photosensitizer was designed to generate singlet oxygen for Photo Dynamic Therapy (PDT) purposes. The ‘PMO’ nanoparticles were shown to be useful for simultaneous imaging and therapy of breast cancer cells under near-infrared (NIR) irradiation. The results highlighted the versatile and useful chemical functionalities, which could be incorporated into ‘PMO’ materials for multiple applications in nanomedicine, including theranostics.

A new family of PMOs containing azobenzene within the pore walls was synthesized by Sayari et al.: bis(4-triethoxysilyl)azobenzene, bis(2,6-dimetyl-4-triethoxysilyl)azobenzene and bis(2,6-diisopropyl-4-triethoxysilyl)azobenzene [36]. The successful formation of the materials was verified by N_2_ sorption, solid-state MAS-NMR spectroscopy and IR spectroscopy. The surface area of the materials ranged from 400–700 m^2^/g. The three materials reveled very different morphology in TEM analysis. Very recently, Haw et al. described the synthesis of nano-sized bis-benzimidazole ‘PMO’, which showed behavior as a ratiometric fluorescence sensors for the detection of Cu^2+^ ions [37]. The new ‘PMO’ was obtained by mixing two Si sources: TEOS and the bis-benzimidazole organic siloxane precursors at different ratios; therefore, it cannot be classified as a true PMO material. In the ‘PMO’, the silica skeleton provides a rigid environment which limits the molecular rotations, resulting in enhanced fluorescence emission. The new ‘PMO’ exhibited dual emission of the enol and keto forms, achieving a ratiometric chemosensor, which showed response to Cu^2+^ ions. The materials showed a very low limit of detection (LOD) of 7.15 × 10^−9^. Inagaki et al. also reported on new PMO possessing molecularly mixed pyridine and benzene moieties in the framework. The results suggested that the pyridine and benzene units are homogenously distributed in the crystal-like pore walls [38]. The fluorescence from the benzene was quenched by the pyridine moieties in the framework. These materials were also obtained at nano-size. They exhibited an ability to absorb Cu^2+^, showing a linear relationship between the loading amount of pyridine moieties and the adsorbed amount of the metal ions. Table 1 overviews the structures of the precursors with aromatic groups, which were used to obtain the different fluorescent PMOs. The abbreviations and names are given if they are used in the literature.

## 3. Luminescence Properties Originating from Lanthanides Grafted or Incorporated into PMOs

The information gathered in the previous section is necessary to understand the background behind the development of lanthanide grafted and incorporated PMOs. It is impossible to talk of lanthanide luminescent PMOs without mentioning that photoluminescent pristine PMOs also exist and a lot of very interesting research has been carried out and is still being carried out on this topic.

This section deals with the reported examples of light emitting lanthanide PMOs. Although there are many lanthanide-based mesoporous silica materials, the amount of actual PMO materials is not that large and it is clearly still a growing field. We hope that with this review, we will interest the readers in this exciting research topic with a wide range of applications.

**Figure 5:**
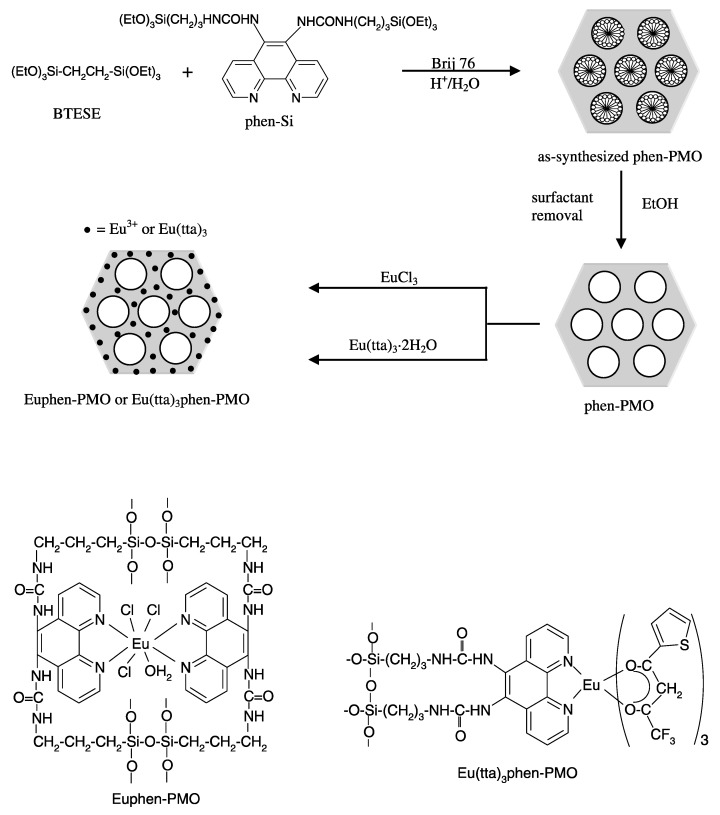
Schematic representation of the co-condensation of 1,2-bis(triethoxysilyl)ethane and 5,6-bis(N-3-(triethoxysilyl)propyl)ureyl-1,10-phenantroline to form a novel PMO material and its post-functionalization with an europium complex [39].

In 2008, Yang et al. reported a new PMO material covalently grafted with 1,10-phenantrolline. This PMO was synthesized via a co-condensation of 1,2-bis(triethoxysilyl)ethane and 5,6-bis(N-3-(triethoxysilyl)propyl)ureyl-1,10-phenantroline (Figure 5) [39].

The PXRD indicated the formation of a well ordered two-dimensional hexagonal structure. Upon increasing the concentration of the phenantroline precursors, the intensity of the (100) reflection decreased gradually, indicating that the ordered assembly of the mesostructure was disturbed. The BET surface area of the PMOs prepared at different precursor ratios ranged from 591 to 1034 m^2^/g. A europium salt (EuCl_3_) and Eu(tta)_3_ complex (tta = thenoyltrifluoroacetone) were covalently grafted into the framework of the PMO in a post-functionalization synthesis procedure. The materials showed the characteristic emission peaks of Eu^3+^: ^5^D_0_→^7^F_0_, ^5^D_0_→^7^F_1_, ^5^D_0_→^7^F_2_, ^5^D_0_→^7^F_3_, ^5^D_0_→^7^F_4_. An enhancement in the luminescence properties was observed for the Eu(tta)_3_phen-PMO materials. A quantum efficiency of up to 27.9% was obtained.

A ‘PMO’ material synthesized through a one-step co-condensation of 1,2-bis(triethoxysilyl)ethane and benzoic acid-functionalized organosilane was reported by Dang et al. [40]. This material belongs to the class of mixed PMOs. FT-IR confirmed the successful incorporation of the benzoic acid into the ‘PMO’ framework. PXRD and N_2_ sorption isotherms revealed the characteristic mesoporous structure with highly uniform pore size distribution. SEM analysis showed that the morphology of the ‘PMO’ was significantly influenced by the ratio of the two organosilica precursors used in the synthesis. TbCl_3_ was grafted onto the ‘PMO’ in an attempt to obtain a luminescent material. This was done through a ligand exchange reaction. It was observed that after TbCl_3_ grafting the mesostructure of the material remained intact. Under UV radiation the characteristic Tb^3+^ emission peaks were recorded: ^5^D_4_→^7^F_6_, ^5^D_4_→^7^F_5_, ^5^D_4_→^7^F_4_, ^5^D_4_→^7^F_3_. When compared with the complex, it was shown that the hybrid material (PMO grafted with Tb^3+^) demonstrated better thermal stability (based on TGA).

Li et al. presented sulfide functionalized Eu/Tb ‘PMO’ hybrids, which exhibited luminescence properties [41]. 4-mercaptobenzoic acid grafted to the coupling agent 3-(triethoxysilyl)propyl isocyanate was used as the precursor for the ‘PMO’ preparation forming a mixed type of PMO. The ‘PMO’ material exhibited three well-resolved diffraction peaks in small-angle XRD, which could be indexed to (100), (110) and (200) reflections associated with 2-D hexagonal symmetry (p6mm), confirming the well ordered mesoporous structure of the material. The Eu- and Tb- grafted ‘PMO’ materials showed nearly unchanged small-angle XRD. The BET surface area of the materials after lanthanide grafting was around 600 m^2^/g. Both the Eu- and Tb- grafted ‘PMO’ materials showed visible emission. However, a clear presence of the host band was present in spectra of both materials, suggesting incomplete energy transfer.

In 2010 Yan et al. published on luminescent lanthanide hybrids covalently bonded to a ‘PMO’ by a calix [4] arene derivative [42]. This material is a mixed PMO as it was prepared by co-condensing Calix-NH_2_-Si (Calix-NH_2_ = 5-amino-25,26,27,28-tetrahydroxycalix [4] arene) with 1,2-bis-(triethoxysilyl)ethane. In this work, a novel bpy-Calix-NH_2_-PMO was prepared by introducing Ln^3+^ (Ln = Eu,Tb) and 2,2′-bipyrdine into the Calix-NH_2_-PMO material. The Calix-NH_2_-PMO material showed good uniformity in the mesostructure and high surface area (687 m^2^/g). The Tb^3+^ grafted material showed very good luminescence properties, whereas the Eu^3+^ material showed the presence of the material’s emission in the emission spectrum, indicating incomplete energy transfer and suggesting that the position of the triplet level of the hybrid material is more favorable for Tb^3+^ excitation. The decay time of the bpy-Tb-Calix-NH_2_ material was 0.616 ms, the lifetime of the Tb-Calix-NH_2_-PMO material was 0.473 and the lifetime of the bpy-Tb-Calix-NH_2_-PMO material was 0.574 ms. Compared with the PMO covalently bonded with the binary complex, the introduction of the second ligand (2,2′-bipyridine) into the mesoporous structure results in more efficient luminescence.

**Figure 6:**
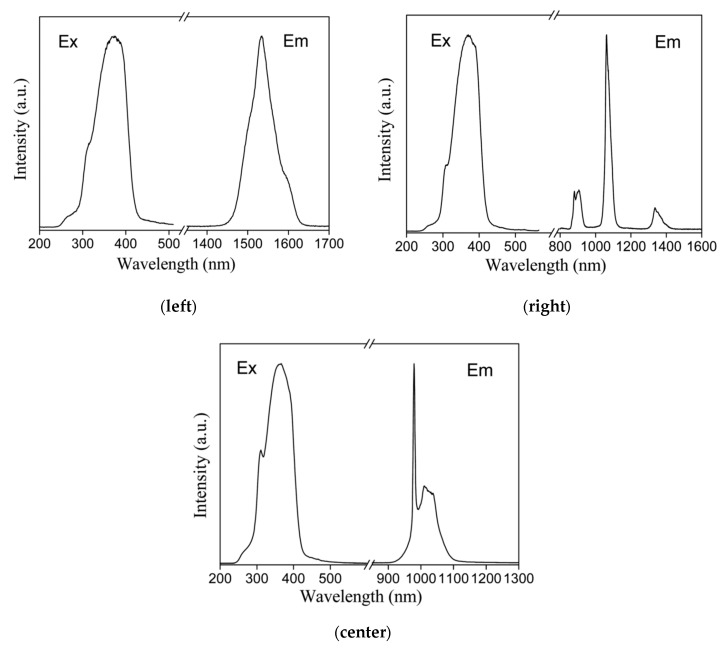
Normalized excitation and emission spectra of the Er^3+^ (**left**), Nd^3+^ (**center**) and Yb^3+^ (**right**) 2,2′-bipyridine-based ‘PMO’ grafted with the appropriate lanthanide complexes [43].

In 2012, a near-infrared emitting lanthanide ‘PMO’ material was reported [43]. In the work by Zhang et al., a 2,2′-bipyridine-based ‘PMO’ was prepared, which, by definition, is a mixed PMO material as the new ‘PMO’ was synthesized though co-condensation of bis(triethoxysilyl)ethane and a pre-synthesized silsesquioxane precursor–4,4′-bis(Si(OEt)_3_(CH_2_)_4_]-2,2′-bipyridine. The ‘PMO’ could be obtained in the form of spherical particles. After introducing lanthanide β-diketonate complexes, Ln(dbm)_3_ the mesostructure of the ‘PMO’ was preserved. After grafting of Nd(dbm)_3_, Er(dbm)_3_ and Yb(dbm)_3_ complexes onto the ‘PMO’, upon excitation with visible light, the hybrid materials showed the characteristic emission peaks for these lanthanides (^4^F_3/2_→^4^I_9/2_, ^4^F_3/2_→^4^I_11/2_, ^4^F_3/2_→^4^I_13/2_ for Nd^3+^, ^4^I_13/2_→^4^I_15/2_ for Er^3+^, and ^2^F_5/2_→^2^F_7/2_ for Yb^3+^). The luminescence decay times were calculated to be 2.37 µs for the Er-‘PMO’ material, 0.095 µs for the Nd-‘PMO’ material and 5.97 µs for the Yb-‘PMO’ material. These good NIR-luminescence properties of the ‘PMOs’ showed a new possibility to use these materials for NIR luminescent applications (Figure 6).

Kim et al. prepared a luminescent biphenylene-bridged ‘PMO’ grafted with Ln^3+^ ions (Ln = Eu, Gd, Tb, Er, Yb) in the presence of 2-thenoyltrifluoroacetone [44]. This is a mixed PMO material. The 4,4′-biphenylene-bridged ‘PMO’ was prepared according to the procedure of Inagaki et al. [9]. Then, a silylation reaction of the 2-thenoyltrifuoroacetone (TTA-Si) was carried out and finally, the hybrid Ln(TTA-Si)_3_(TTA-Si_2_)Bp-PMO materials were developed. Despite being referred to as a ‘PMO’ in the publication, this complex material lacked mesoscale periodicity, although the starting material showed a hump at 2θ = 1.88° and also five distinct peaks at 2θ = 7.42, 15.02, 22.48, 30.1 and 37.8°. The presence of the signal at 2θ = 1.88° corresponding to a d spacing of 44 Å is indicative of the mesoscopically ordered biphenylene-bridged ‘PMO’. TEM and SEM revealed that the ‘PMO’ consisted of primary particles approximately 100–200 nm in size. The uniform mesochannels in a hexagonal arrangement and molecular-scale periodicity were clearly observed in the TEM image of the pristine PMO. A Gd-grafted (TTA-Si)_3_(TTA-Si_2_)Bp-PMO material allowed the determination of the triplet level of the material at 77 K. It was calculated to be around 450 nm which is equal to 22 2000 cm^−1^. The Ln(TTA-Si)_3_(TTA-Si_2_)Bp-PMO materials were investigated for their visible (Eu, Tb) and NIR (Yb, Er) luminescence. The authors showed that the emission color of Ln/Ln’ mixed metallic hybrids could be tuned by varying the properties of the two metal ions. The Eu^3+^ and Tb^3+^ emission increased upon co-grafting the ‘PMO’ hybrid materials with Gd^3+^ ions. In the case of Eu/Tb co-grafted material quasi-white light could be obtained. The intrinsic QYs and room temperature lifetime values obtained for Eu-containing hybrid ‘PMO’ materials were either comparable or slightly larger than those reported for other Eu organic-inorganic hybrids based on tris-2-thenoyltrifluoroacetonate complexes. In general, the QYs were not high but these kinds of hybrid materials provide, without a doubt, other advantages, such as thermal stability and processability.

A white-light emitting hybrid ‘PMO’ was developed based on a phenanthroline functionalized PMO, Eu^3+^ ions and 2-methyl-9-hydroxyphenalenone [45]. The ‘PMO’ precursors were prepared from 5-amino-1,10-phenantroline and 3-(triethoxysilyl)propylisocyanate. As this material is partially built out of TEOS, it is not a true PMO material. The resulting hybrid ‘PMO’ material Eu(MHPO)_3_phen-PMO(NO_3_)_3_ (MHPO = 2-metyl-9-hydroxyphenalenone) displayed a type IV isotherm with H1-type hysteresis loops at high relative pressure according to the UIPAC classification. The BET surface area was determined to be 401 m^2^/g and the pore size was 2.09 nm. In the excitation spectrum, a wide excitation band ranging from 220 to 500 nm was observed (from UV region to violet-blue visible region). The material did not exhibit completely efficient energy transfer to the Eu^3+^ ions, which resulted in the presence of both the red emission of the Eu^3+^ as well as the blue emission of the ‘PMO’ host in the emission spectrum. This strategy allowed the realization of white-light emission from a ‘LnPMO’ hybrid system.

**Figure 7:**
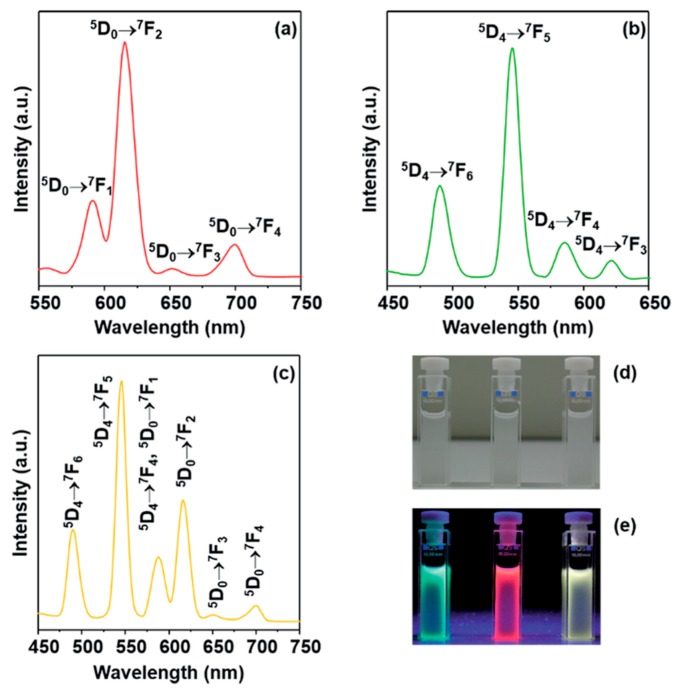
Emission spectra of (**a**) Eu-grafted DPA-‘PMO’, (**b**) Tb-grafted DPA-‘PMO’ and (**c**) Eu/Tb-grafted DPA-‘PMO’. Images of the three samples under sunlight (**d**) and under a UV lamp (**e**) [46].

In 2015, Anwander et al. communicated on the nano-sized ‘PMO’ obtained via co-condensation of N,N-bis(trimethoxysilylpropyl)-2,6-pyridine dicarboxoamide with tetraethylorthosilicate (TEOS) (DPA ‘PMO’) [46]. This material, according to the strict definition, does not classify as a PMO material as it is partially built out of TEOS. The formation of the ‘PMO’ was confirmed through FT-IR and ^13^C and ^29^SI MAS NMR spectroscopy. This ‘PMO‘ showed spherical morphology (particles around 70 nm in diameter). PXRD confirmed the formation of an ordered mesostructure. The surface area of the ‘PMO’ fluctuated a bit depending on the ration of the precursors used. For example when 5% N,N-bis(trimethoxysilylpropyl)-2,6-pyridine dicarboxoamide was employed, the BET surface area was 921 m^2^/g. Furthermore, in the work, EuCl_3_, TbCl_3_ and a mixture of EuCl_3_ and TbCl_3_ were grafted onto the ‘PMO’. A small drop of BET surface area (ca. 870–860 m^2^/g) was observed, confirming the successful grafting of the lanthanides. The material formed a stable milky suspension when dispersed in water, which, under UV irradiation, exhibited the characteristic red (Eu-‘POM’), green (Tb-‘POM’) and yellow (Eu,Tb-‘POM’) emission (Figure 7). It is worth adding that in TGA, it was observed that the onset of decomposition for the lanthanide-grafted ‘PMOs’ (360 °C) was at higher temperatures that that of the parent ‘PMO’ (330 °C), which, once again, shows that incorporating lanthanide ions into a ‘PMO’ material improves its thermal stability.

**Figure 8:**
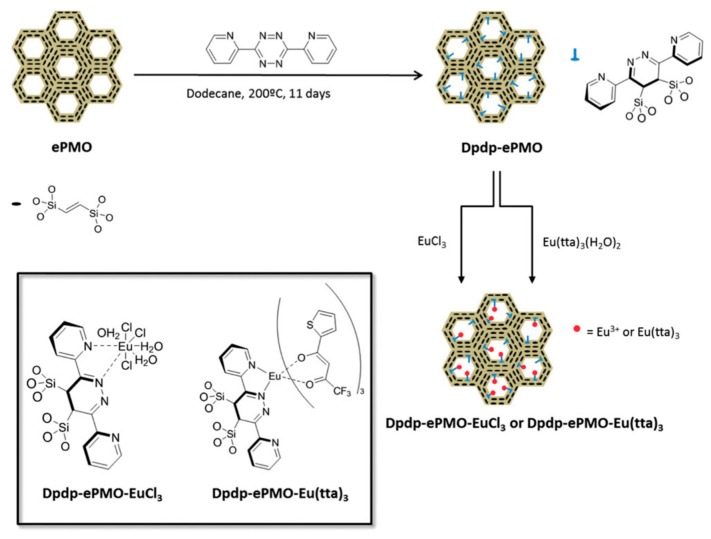
Schematic overview of the synthetic procedure for the formation of the surface Diels-Alder adducts on the ePMO and predicted structure of the resulting organic-inorganic luminescent hybrid mesoporous materials grafted with europium ions or complexes [47].

In 2015, Van Der Voort et al. reported on an ethenylene-bridged PMO (ePMO) which was decorated in its pores with dipyridyl-dihydropyridazine units [47]. This was done through a hetero Diels-Alder reaction between the double bond of the ePMO and a substituted tetrazine. The successful formation of the surface Diels-Alder adduct was proven by the presence of new signals in the aromatic region in ^13^CP/MAS NMR. The BET surface area of the parent PMO changed from 832 m^2^/g to 448 m^2^/g after lanthanide grafting. Moreover, the pore size decreased from 6.3 Å to 5.1 Å, confirming the successful grafting of the lanthanide in the pores. As a proof of principle, the material was grafted with EuCl_3_ and Eu(tta)_3_ (Figure 8). This allowed the development of two new organic-inorganic mesoporous luminescent hybrid materials. The PMO hybrid material grafted with Eu(tta)_3,_ showed stronger luminescence properties, indicating that tta worked as a second “antenna” ligand in the system. It reached a QY of 7.5% and an average decay time of 0.385 ms.

Chen et al. prepared novel rattle-structured upconverting luminescent materials by growing a organosilica shell around β-NaLuF_4_:Gd,Yb,Er [48]. The ‘PMO’ outer shell was prepared through hydrolysis of 1,4-bis(triethoxysilyl)benzene and 3-aminipropyl triethoxysilane. The shell thickness was estimated to be around 10 nm around β-NaLuF_4_ particles which were around 20 nm in size. The particles had a void between the β-NaLuF_4_ particles and PMO shell obtained through SiO_2_ shell etching. The void was used to load with photosensitizer and tested for NIR-triggered upconversion induced Photo Dynamic Therapy (PDT). The biological safety of the material was demonstrated in vitro.

In 2016, Balula et al. reported on the first red emitting polyoxometalate@PMO material [49]. A ethylene-bridged amine-functionalized PMO was first prepared and further [Eu(W_5_O_18_)_2_]^9−^ polyoxometalate (POM) was incorporated into the composite. Extensive analysis revealed incorporation of the EuPOM within the PMO framework with morphological preservation of the mesoporous organosilica in the final composite material. The incorporation of the EuPOM in the PMO led to the formation of a strongly red emitting material upon UV irradiation. Interestingly, a change in the coordination environment of the Eu^3+^ ions was observed after incorporation into the PMO framework. It was proven that the [Eu(W_5_O_18_)_2_]^9−^ POM breaks apart after reacting in solution for 24 h with the PMO material. Additionally, it was presented that the interaction of the EuPOM with the amine groups causes strong distortion of the Eu^3+^ site symmetry. These two factors led to quite significant differences in the luminescence properties of the EuPOM and EuPOM incorporated in the PMO framework.

Chen et al. proved that luminescent PMOs based on lanthanides could also be prepared using a completely different approach [50]. In their work, they employed a “ship-in-a-bottle” approach to construct PMOs with lanthanide nanoparticles built inside of them. The spherical ‘PMO’ was obtained by reacting 1,4-bis(triethoxysilyl)benzene with 3-aminopropyltriethoxysilane. They were grown around a silica template, which was later etched out to create hollow ‘PMO’ spheres. Upconverting fluoride particles of NaYF_4_:Yb,Er, NaLuF_4_:Yb,Er, NaGdF_4_:Yb,Er and LiYF_4_:Yb,Er were grown inside the hollow void, creating a new type of hybrid organic-inorganic materials. These materials showed strong green upconversion luminescence upon 980-laser excitation. After loading with doxorubicin (DOX), they could be used for controlled drug release by utilizing the energy transfer from the upconversion fluorides to DOX.

A novel Schiff-based derived ‘PMO’ was communicated by Wang et al. [51]. The Salen-‘PMO’ was synthesized through the co-condensation of 1,2-bis(triethoxysilyl)ethane and modified Salen-type Schiff-base compound N,N’-bis(salicylidene)ethylenediamine in the presence of a template (Pluronic P123). N,N’-bis(salicylidene)ethylenediamine grafted on the coupling agent 3-(triethoxysilyl)-propyl-lisocyanate was used as the ‘PMO’ precursor. Small angle XRD indicated that a highly ordered two-dimensional (2D) material with hexagonal symmetry was formed. After grafting Eu^3+^ and Tb^3+^ onto the ‘PMO’, it was observed that the Bragg peak indexed as (100) decreased in intensity. The authors explained that the decrease in intensity is most likely due to X-ray absorption/scattering by the Ln^3+^ ions. N_2_ sorption measurements reveled that all the materials exhibited a type-IV isotherm curve with H1-type hysteresis loops at a relative pressure, which is typical of conventional mesoporous materials prepared in the presence of this type of surfactant. The BET surface area of the pristine material was 828 m^2^/g; however, it dropped after lanthanide grafting to around 200 m^2^/g, proving the successful grafting. Moreover, a drop in the pore diameter was recorded. TEM images demonstrated p6mm symmetry of the materials. The lanthanide grafted materials showed the characteristic emission peaks of Tb^3+^ and Eu^3+^. The energy transfer and emission intensity was strongly enhanced when a second co-ligand was added: 1,10-phenanotroline. The quantum efficiency of the materials also strongly increased after including the second co-ligand. For example, for the Eu-grafted Salen-‘PMO’, a quantum efficiency of 5.74% was obtained. When the phenanthroline ligand was added to the materials, it increased to 51.35%. The authors explained that the reason for this increase is quantum efficiency is because the substitution of the silanol with covalently bonded phenanthroline groups in the pore channel of the mesoporous ‘PMO’ host material decreases the level of nonradiative multiphonon relaxation by coupling to –OH vibrations and nonradiative transition states.

In the work of Van Deun et al., the ePMO material was investigated again [52]. It was functionalized with dipyridyl-pyridazine (dppz) units and then further grafted with NIR emitting lanthanide ions (Nd^3+^, Er^3+^ and Yb^3+^). The parent PMO material was prepared by hetero Diels-Alder reaction between 3,6-di(2-pyridyl)-1,2,4,5-tetrazine and the double bond of the ePMO. The same reaction was carried out for vinyl-silica and the materials were compared in this study. Two types of lanthanide complexes were grafted onto the materials: 2-thenoyltrifluoroacetone or benzoyltrifluoroacetone. They were selected due to their triplet level matching well with the accepting levels of NIR lanthanides. It should also be mentioned that the dppz unit has a low triplet level, which makes it suited for coordinating with NIR lanthanides. Although the lanthanide grafted vinyl-silica material was easier and faster in preparation, the advantage of the lanthanide grafted ePMO material was its known higher stability originating from the PMO. Detailed luminescence studies, including temperature-dependent investigations, were carried out. These materials could be successfully excited through the broad ligand band as well as directly through the sharp f–f transitions. The Yb^3+^ materials were evaluated for their potential use as ratiometric thermometers in the 110–310 K temperature range.

**Figure 9:**
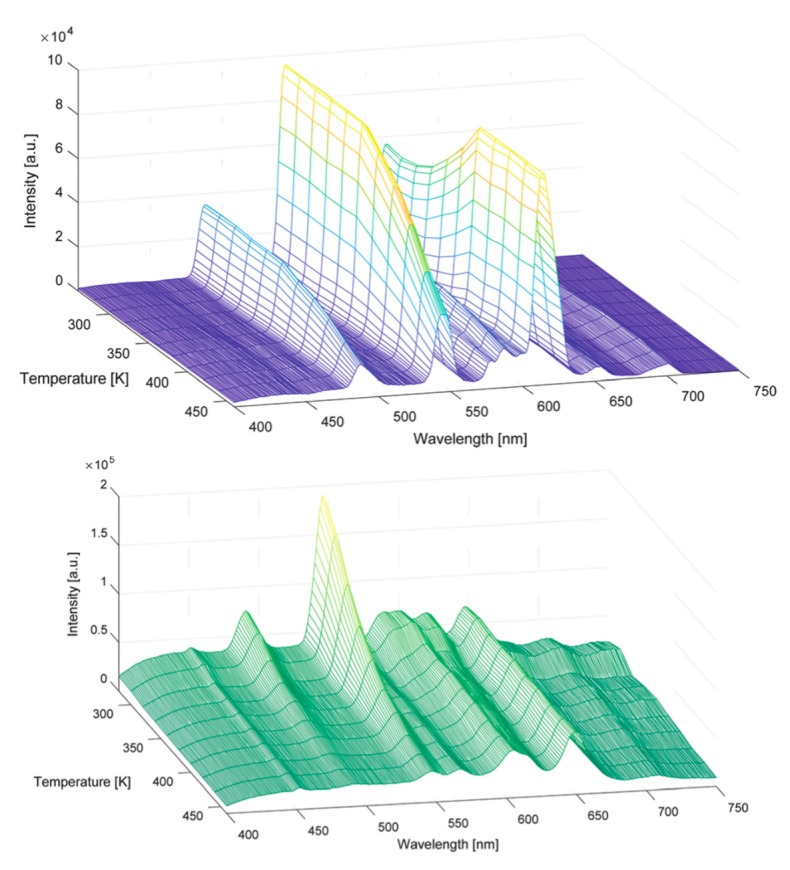
(**Top**): Emission maps of the spectra recorded at 260–460 K for a Eu,Tb co-grafted DPA-PMO material. (**Bottom**): Emission maps of spectra recorded at 280–460 K for a Sm,Tb co-grafted DPA-PMO material [53].

In 2019, Van Der Voort et al. once again reported on the ‘PMO’ prepared from 5% N,N-bis(trimethoxysilylpropyl)-2,6-pyridine dicarboxoamide precursor and 95% TEOS [53]. As mentioned earlier in the text, this is not a true type of PMO as it contains TEOS. This time, this highly versatile ‘PMO’ was used for grafting Tb/Eu and Tb/Sm with 1,10-phenanotroline as a co-ligand and was tested for its use as a biological nanothermometer. Many materials have already been used for the preparation of nanothermometers, especially nanoparticles of inorganic phosphors and nano-MOFs; however, nano-PMOs were not considered for this application until then [54,55,56]. The regular nano-sized morphology (around 50–70 nm spheres) of the ‘PMO’ material and very strong visible luminescence made it an ideal candidate for this application. Several different materials with varied ratios of Tb/Eu and Tb/Sm were prepared to be tested for potential thermometer applications. The Eu,Tb-grafted ‘PMO’ materials showed good sensing capability in the 260–460 K range. The Sm,Tb-grafted ‘PMO’ materials also showed good sensing capability in the 280–460 K temperature range (Figure 9). A very good relative sensitivity of 2.3807%K^−1^ was obtained for a Sm,Tb-grafted ‘PMO’ material. It should be mentioned that Sm,Tb-based thermometers are rather rare in the literature as it is generally difficult to obtain strong Sm^3+^ emission, especially at elevated temperatures. The good performance of these materials as optical thermometers in the physiological range (and beyond that too), as well as the nano-size and good biocompatibility of ‘PMO’ materials makes them attractive materials to further study for potential use as biological nanothermometers. For more information on the biocompatibility abilities of PMOs, the reader is referred to [6,57,58].

**Figure 10:**
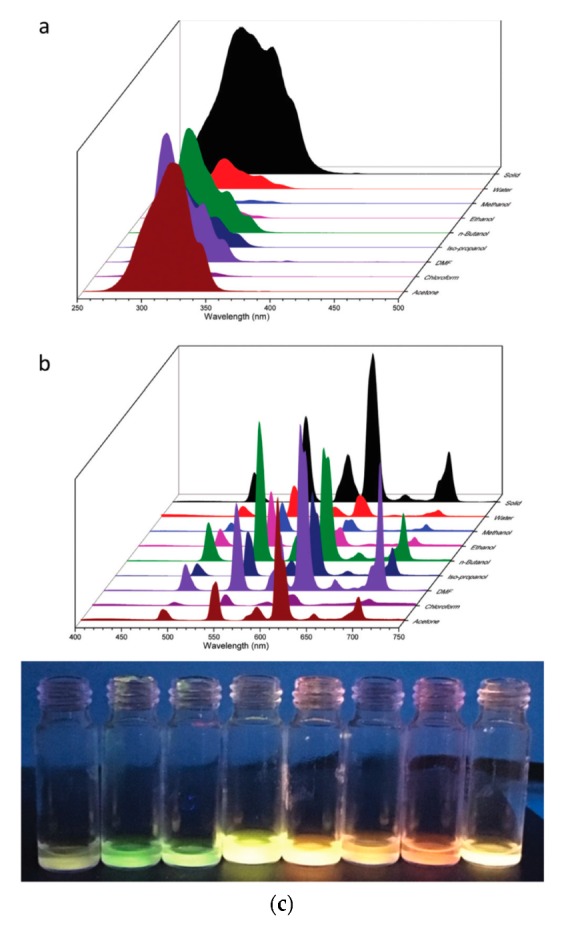
(**a**) Excitation spectra and (**b**) emission spectra of the DPA-‘PMO’ grafted with Eu,Tb and 1,10-phenanotroline in solid state and dispersed in different solvents. (**c**) Photo taken under a UV lamp (302 nm excitation) of the ‘PMO’ hybrid material in the different solvents (from left to right: water, methanol, ethanol, n-butanol, iso-propanol, DMF, chloroform and acetone) [59].

Shortly after, the same research group employed the same nano-sized ‘PMO’ to show the possibility of using ‘LnPMOs’ as chemical sensors both for ion and solvent sensing [59]. As it was mentioned in the previous section, pristine aromatic containing ‘PMOs’ have previously been tested for ion sensing ability based on fluorescence change and showed very promising behavior. An interest developed in employing ‘LnPMOs’ also for chemical sensing. The so-called DPA-‘PMO’ (synthesized from 5% N,N-bis(trimethoxysilylpropyl)-2,6-pyridine dicarboxoamide precursor and 95% TEOS) was grafted with Eu^3+^, Tb^3+^ or a 50:50 mixture of Eu^3+^ and Tb^3+^. Two different co-ligands were additionally used–1,10-phenantroline and 5,5′-dimethyl-2,2′-dipyridyl. In the study, standard solutions containing 10 ppm nitric acid metal salts (Hg^2+^, Zn^2+^, Cu^2+^, Ca^2+^, Cr^3+^, Fe^2+^, Mn^2+^, and Pb^2+^) were used. It was observed that the lanthanide ion had no influence on the selective ion sensing behavior. This may, however, be connected to the fact that the studied Eu^3+^ and Tb^3+^ are quite similar in size. On the other hand, the presence or absence of the second co-ligand played a significant role in the ion sensing behavior and selectivity. The materials without a co-ligand showed no specific selectivity for any of the metal ions. However, the materials with a co-ligand showed strong “turn on” fluorescence for Pb^2+^ and Cr^3+^ ions. Some selectivity was also observed for the highly toxic Hg^2+^ ions. The hybrid materials containing the two different co-ligands showed very similar sensing performance. A more detailed investigation indicated that most likely, a dynamic quenching mechanism was responsible for the reduction in the luminescence intensity of the ‘PMO’ hybrid materials in the presence of certain metal ions. The enhancement of the luminescence in the presence of Pb^2+^, Cr^3+^ and Hg^2+^ ions can most likely be linked to a strong antenna effect from the second co-ligand after their formation of complexes with the metal ions. Therefore, these materials showed promising behavior for selective ion sensing in solutions. Additionally the mixed Eu,Tb materials were studied for their solvatochromic behavior and ability to be used for solvent sensing. All the investigated materials, both with and without co-ligand, showed significant solvatochromism, which was caused by the change in the Tb-to-Eu ratio of the emission peaks. This suggests that a change in the Tb-to-Eu energy transfer takes place in the presence of different protic and aprotic solvents (Figure 10).

**Figure 11:**
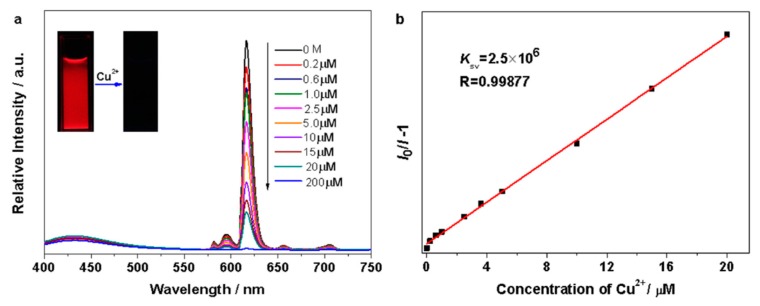
(**a**) The photoluminescence spectra of the PMO prepared through the co-condensation of 1,2-bis(triethoxysilyl)ethane and modified 4′-(4-carboxy-methyleneoxy phenyl)-2,2′:6′,2”-terpyridine in Cu(NO_3_)_2_ aqueous solution at different concentrations (excited at 333 nm). The inset shows changes in luminescence following the addition of Cu^2+^ ions (2 x 10^−4^ M) to the Eu^3+^ grafted PMO suspension under UV light (365 nm). (**b**) Stern–Volmer plot for the PMO hybrid material sensing of Cu^2+^ ions at the range of 0-2 x 10^−5^ M in aqueous solution [60].

The interest in using lanthanide PMO materials for ion sensing was also expressed by the group of Yu who published highly selective luminescent sensing of Cu^2+^ ions in aqueous solution, employing a Eu-grafted PMO [60]. The PMO used in this study was prepared through co-condensation of 1,2-bis(triethoxysilyl)ethane and modified 4′-(4-carboxy-methyleneoxy phenyl)-2,2′:6′,2”-terpyridine in the presence of Pluronic P123. It is known that the terpyridine moiety forms interesting chelates with Eu^3+^ ions. An interesting new hybrid material was obtained by linking the PMO with a europium β-diketonate complex. In the PXRD, the materials showed a strong reflection peak (100) at low angle 2θ and two short second-order reflection peaks (110) and (200), characteristic of hexagonal arrangement of the uniform pores in the materials. For the lanthanide grafted material, the Bragg peak intensity dropped as was observed in previously reported lanthanide-grafted materials. TEM images confirmed very good ordering of the materials. The BET surface area of the PMO dropped as observed in all other cases after lanthanide grafting. Upon UV irradiation, the materials showed a strong red emission color and the characteristic emission peaks of the Eu^3+^ ion. This new hybrid material was also tested for its ability as a chemosensor. It showed selective sensing of Cu^2+^ ions in aqueous solution with a quenching efficiency K_sv_ = 2.5 × 10^6^ (Figure 11).

In very recent work, Van Der Voort et al. communicated on an amine-containing mixed ‘PMO’, which could be obtained from a long linear amine precursor (3-trimethoxysilylpropyl)diethylenetriamine at 5% or 10% and 1,2-bis(triethoxysilyl)ethane [61]. This ‘PMO’ was very easy to synthesize and it was shown that it could be used for a wide range of applications, including, among others, luminescence- and luminescence-based ion sensing. The formation of the ‘PMO’ was confirmed using techniques such as PXRD, N_2_ sorption and ^13^C CP/MAS NMR. After the preparation of the ‘PMO’, the dangling amine groups were grafted with a aldehyde to form Schiff base ligand functionalized luminescent hybrid ‘PMO’ materials. These Schiff base ligand functionalized ‘PMOs’ alone already showed luminescent properties with a maximum emission at around 550 nm. These ‘PMOs’ were subsequently reacted with lanthanide salts or lanthanide complexes yielding novel luminescent hybrid ‘PMO’ materials. These ‘PMO’ materials could also be prepared at nano-size, yielding small spheres 20 nm in size. A material obtained from grafting the ‘PMO’ with o-vanilline and further post-grafting with Eu(tfac)_3_ complex (tfac = 1,1,1-trifluoroacetylacetone) showed the strongest luminescence properties and was further used for ion sensing studies. The material showed significant “turn on” fluorescence in the presence of Hg^2+^ ions.

**Figure 12:**
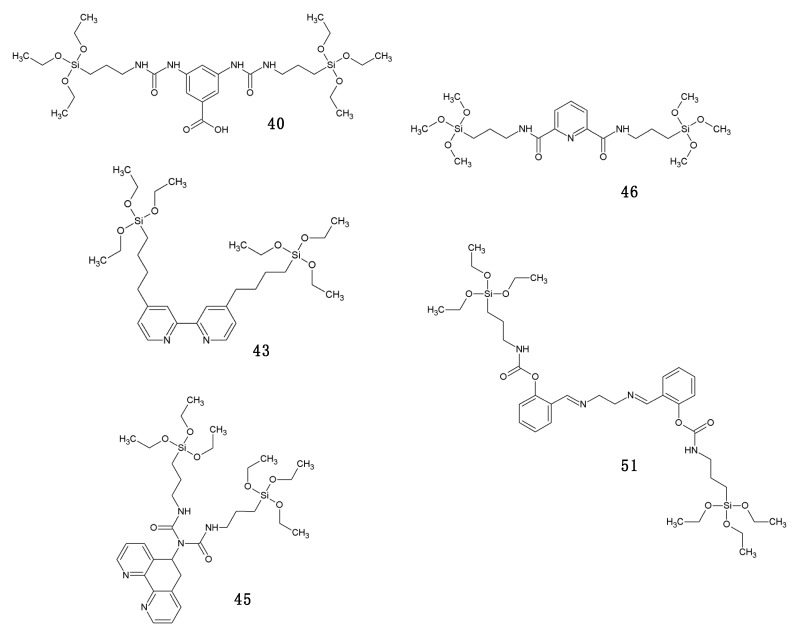
Overview of selected PMO precursors used for the formation of LnPMOs. The reference to the appropriate publication is given under the precursor structure.

Figure 12 overviews some of the PMO and ‘PMO’ precursor structures, which were used for developing LnPMOs/’LnPMOs’. Table 2 summarizes the proposed applications of the luminescent LnPMO/’LnPMOs’ materials. Throughout the review, it can be seen that various co-ligands are used to functionalize the LnPMO’LnPMOs’ materials. They are overviewed in Table 3, with a mention of relevant features of this group.

## 4. Conclusions

In this review, we presented the current literature concerning lanthanide PMO and ‘PMO’ materials, with an introductory section on aromatic PMOs, which can emit fluorescence without any post-grafting of metals, as an extended overview of the field. Currently, it is most common to graft lanthanide salts or complexes onto the aromatic framework of PMOs, which work as an antenna for luminescence. However, some recent reports have already proposed that PMOs can be used as a biocompatible shell grown around inorganic nanoparticles. A wide range of PMO hybrid organic-inorganic materials has already been proposed, yet there are still many possibilities for developing novel materials. A trend that can be noticed is that an interest in nano-sized PMOs has emerged in recent years. This is especially important for future biological applications of the materials. Moreover, in recent years, new, concrete applications of the LnPMO materials have been proposed; for example, for (nano) thermometry, ion sensing, solvent sensing, Photo Dynamic Therapy and drug delivery. Throughout this review we tried to give a concise overview of the field in the hope to interest the reader in these exciting materials with many promising applications.

## Figures and Tables

**Table 1:** Overview of the different aromatic precursors used to obtain PMO materials (* abbreviation and name given if available in the literature).

Abbreviation *	Full Name *	Structural Formula	Ref. in Text
BTEB	1,4-bis(triethoxysilyl)benzene	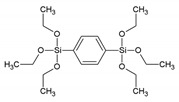	[8]
BP	4,4′-bis(triethoxysilyl) -biphenyl	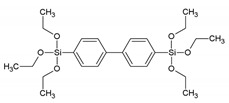	[9]
-	1,4-bis(triethoxysilylmethyl)benzene	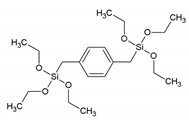	[10]
BTMEB	1,4-bis(trimethoxysilylethyl)benzene	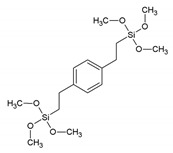	[10]
BTEVB, vPh	1,4-bis-((*E*)-2-(triethoxysilyl)vinyl)benzene	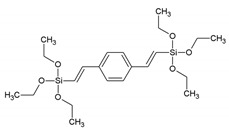	[11,38]
BTET	2,5-bis- (triethoxysilyl)thiophene	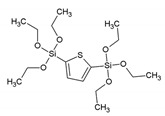	[12]
-	-	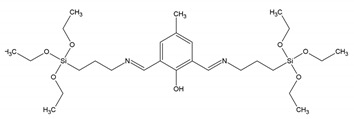	[14]
-	9,10-bis-{4-[2-(3-trimethoxysilylpropylthio)-ethyl]phenyl}anthracene	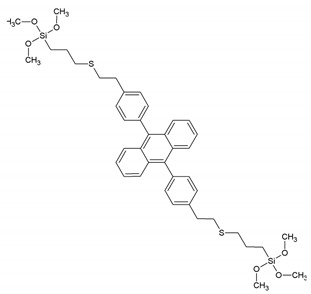	[15]
-	-	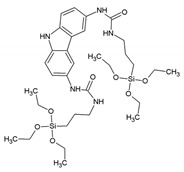	[16]
BTEVS	4,4′-bis((*E*))-2-(triethoxysilyl)vinyl)stilbene	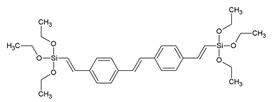	[17]
BTEVAB	1,2-bis(4-((*E*)-2-(triethoxysilyl)vinyl)phenyl)diazene	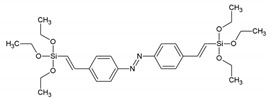	[17]
Nph/BTENph	2,6-bis(triethoxysilyl)naphthalene	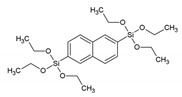	[18]
Ant/9,10- BTEANT	9,10-Bis(triethoxysilyl)anthracene	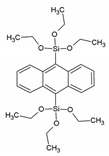	[18]
PTCDBS	-	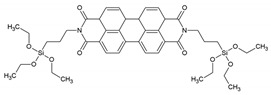	[19]
2,6-BTEAnt	2,6-bis(triethoxysilyl)anthracene	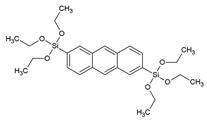	[21]
BTEAD	2,7-bis(triethoxysilyl)acridone	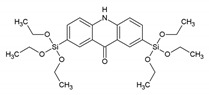	[23]
Phen-Si	-	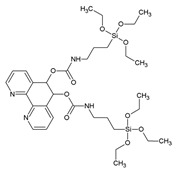	[32]
EB	-	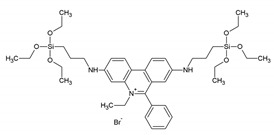	[33]
TPE-Si	-	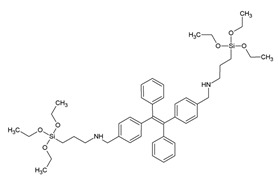	[34]
N2F	-	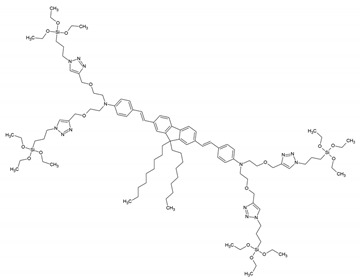	[35]
-	bis(4-triethoxysilyl)azobenzene	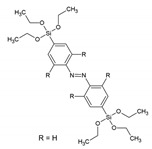	[36]
-	bis(2,6-dimetyl-4-triethoxysilyl)azobenzene	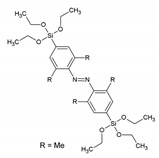	[36]
-	bis(2,6-diisopropyl-4-triethoxysilyl)azobenzene	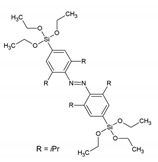	[36]
BBM-Si	-	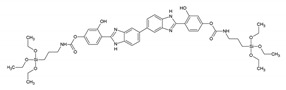	[37]
vPy	2,5-bis[(*E*)-2-(triethoxysilyl)vinyl]pyridine	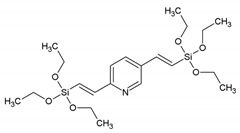	[38]

**Table 2:** Summary of the proposed applications for the LnPMO materials.

Application	Incorporated Lanthanide(s)	Ref. in Text
Visible emission	Eu^3+^	[39,44,46,47,49,51]
	Tb^3+^	[40,44,46,51]
	Eu^3+^/Tb^3+^	[41,42,46]
	Gd^3+^	[44]
Near-infrared emission	Er^3+^	[43,44,52]
	Nd^3+^	[43,52]
	Yb^3+^	[43,52]
White light emission	Eu^3+^/Tb^3+^	[44]
	Eu^3+^	[45]
Upconversion induced Photo Dynamic Therapy	Er^3+^/Yb^3+^	[48]
Upconversion induced drug delivery	Er^3+^/Yb^3+^	[50]
(Nano)thermometry	Er^3+^, Nd^3+^, Yb^3+^	[52]
	Tb^3+^/Eu^3+^, Tb^3+^/Sm^3+^	[53]
Ion sensing	Eu^3+^	[59,60,61]
	Tb^3+^	[59]
Solvent sensing	Tb^3+^/Eu^3+^	[59]

**Table 3:** Summary of the different organic moieties used to functionalize the LnPMOs.

Class of Organic Group	Organic Group	Employed Lanthanide	Relevant Features	Ref.
β-diketonates	Thenoyltrifluoro- acetone (tta)	Eu^3+^	Provides two oxygen coordinating sites; enhances the photoluminescence compared to lanthanide salt	[39,47]
		Eu^3+^, Gd^3+^, Tb^3+^, Er^3+^, Yb^3+^	tta is grafted onto the biphenylene-bridged PMO through silylation reaction; provides two oxygen coordination sites	[44]
		Nd^3+^, Er^3+^, Yb^3+^	Provides two oxygen coordinating sites; enhances the photoluminescence compared to lanthanide salt; triplet level suitable for NIR lanthanides	[52]
	Dibenzoylmethane (dbm)	Nd^3+^, Er^3+^, Yb^3+^	Provides two oxygen coordinating sites; low triplet level suitable for NIR emitting lanthanides	[43]
	Benzoyltrifluoro- acetone (bta)	Nd^3+^, Er^3+^, Yb^3+^	Provides two oxygen coordinating sites; enhances the photoluminescence compared to lanthanide salt; triplet level suitable for NIR lanthanides	[52]
	1,1,1-trifluoroacetyl-acetone (tfac)	Eu^3+^	Provides two oxygen coordinating sites; enhances the photoluminescence compared to lanthanide salt	[61]
Heterocyclic nitrogen donors	Dipyridyl- pyridazine (dppz)	Eu^3+^	Grafted onto ePMO through Diels-Alder reaction; provides two nitrogen sites to coordinate a lanthanide to the PMO	[47]
		Nd^3+^, Er^3+^, Yb^3+^		[52]
	1,10-Phenanotroline (phen)	Eu^3+^, Tb^3+^	Two nitrogen coordination sites provided; the energy transfer and emission intensity, QY and decay were strongly enhanced when the second co-ligand was added; significant influence on ion sensing and solvatochromic behavior	[51]
		Eu^3+^/Tb^3+^		[53,59]
		Sm^3+^/Tb^3+^		[53]
	2,2′-Bipyrdine (bpy)	Eu^3+^, Tb^3+^	Coordination through two nitrogen sites; enhancement in emission intensity and decay compared to lanthanide salt	[42]
	5,5′-Dimethyl-2,2′-dipyridyl (bpy)	Eu^3+^/Tb^3+^	Coordination through two nitrogen sites; enhancement in emission intensity and decay compared to lanthanide salt; significant influence on ion sensing and solvatochromic behavior	[59]
Heterocyclic oxygen donors	2-Metyl-9-hydroxyphenalenone (MHPO)	Eu^3+^	Coordinated through two oxygen atoms	[45]
Schiff base forming ligands	o-Vanilline (o-van)	Eu^3+^	Forms Schiff base bind with linear amine dangling on PMO; provide coordination environment for lanthanide ions	[61]
